# Urinary Paraben Concentration and Its Association with Serum Triglyceride Concentration in 2013-2014 NHANES Participants: A Cross-Sectional Study

**DOI:** 10.1155/2020/8196014

**Published:** 2020-09-15

**Authors:** Rebecca Pazos, Cristina Palacios, Adriana Campa

**Affiliations:** Department of Dietetics and Nutrition, Robert Stempel College of Public Health and Social Work, Florida International University, 11200 SW 8th Street, AHC5, Miami, FL 33199, USA

## Abstract

**Background:**

Alkyl esters of para-hydroxybenzoic acid, colloquially known as parabens, are types of preservatives found in multiple foodstuffs, pharmaceuticals, and personal care products to which Americans are exposed daily. It is unclear if parabens exhibit endocrine-disrupting properties. Parabens may interact with triglycerides in adipose tissue and impact lipid metabolism.

**Objective:**

To evaluate the association between urinary paraben concentrations and serum triglyceride concentrations.

**Design:**

A cross-sectional study. *Setting*. The Mobile Examination Centers affiliated with 2013-2014 NHANES. *Participant(s)*. 827 adults (20 years or older) affiliated with the 2013-2014 NHANES. *Intervention(s)*. None. *Main Outcome Measure(s)*. Triglyceride levels were associated with urinary paraben concentrations (methyl, ethyl, and propyl) using a hierarchical multiple regression, adjusting for ethnicity/race, gender, BMI, and age. Unadjusted results are also reported.

**Results:**

The geometric mean of the urinary concentration of methylparaben, ethylparaben, and propylparaben was 57.100, 2.537, and 6.537 ng/ml, respectively. Triglyceride concentrations were inversely associated with methylparaben (*β* = −0.092, *P*=0.07), ethylparaben (*β* = −0.066, *P*=0.045), and propylparaben (*β* = −0.076, *P*=0.025). Being female, non-Hispanic White, and non-Hispanic Black were associated with decreasing triglyceride levels in the presence of methylparaben, ethylparaben, and propylparaben, and age, BMI, and being male were associated with increasing circulating triglycerides.

**Conclusion:**

Despite the potential detrimental effects of parabens on triglycerides, our results suggest that urinary excretions of methylparaben, ethylparaben, and propylparaben are associated with lower concentrations of circulating triglycerides in certain populations. Further research is needed to confirm the mechanisms and health impact of this relationship.

## 1. Introduction

Alkyl esters of para-hydroxybenzoic acid, colloquially known as parabens, are a type of preservative commonly found in foodstuff, pharmaceuticals, and personal care products [1–7]. These compounds can display broad antimicrobial activity, are chemically stable, and are relatively inexpensive to fabricate [7]. However, in recent years, it has been suggested that parabens may exhibit endocrine-disrupting properties similar to bisphenol A, phenols, phthalates, 17 alpha-ethynylestradiol, and cypermeyhrin [1–9]. Due to their possible detrimental health properties, as well as their ubiquitous nature, it is imperative to study how parabens interact with the human metabolism.

There are four main types of parabens that are found in foodstuff, pharmaceuticals, and personal care products: methylparaben, ethylparaben, propylparaben, and butylparaben. Parabens are naturally found in vegetables and fruits; however, they are also found in high concentrations in prepared foods, grains, beverages, and dairy products. Specifically, high concentrations of methylparabens can be found in iced tea, muffins, pudding, and turkey roast [10]. A variety of red wine has a significant amount of ethylparaben, and propylparaben is found in smaller concentrations in food [10]. In terms of medication, fluoxetine, ibuprofen, and diphenhydramine contain methylparaben, propylparaben, and butylparaben and dextromethorphan and guaifenesin contain methylparaben and propylparaben [11]. Additionally, methylparaben and propylparaben have been detected in urine samples of people who ingested the above medications even 26 hours after the medication has been taken; this is surprising because parabens have a relatively short half-life [1, 11]. In addition to urine, parabens have often been detected in human cells, milk, and tissue samples [1–5]. Several studies have associated urinary paraben levels with personal care product usage [1–3]. Furthermore, a study conducted in trauma victims found methylparabens in the bulk of their urine and serum samples [5]. Specifically, this study discovered methylparabens in 40% of their adipose tissue samples and concluded that these paraben levels did not correlate with the urine or serum samples [5].

Molecular studies on parabens have provided some evidence on how parabens may be interacting with various cells. An *in vitro* study conducted in mesenchymal stem cells suggests that butylparabens modulate their fate towards adipocytes [6]. A study conducted on mammalian and bacterial lipid monolayers suggests that the influence of parabens on mammalian cells is based on the chemical structure of the paraben, the specific class of the membrane, and the concentration of the paraben solution [7]. This study also provides evidence that butylparabens can modulate lipid films by changing the orientation of the lipid molecule, which reduces their intermolecular stability [7]. Further, parabens can interface with mammalian and bacterial lipids with the former being more susceptible to this interaction because mammalian cells lack a cell wall [7].

Animal studies have generated additional evidence that endocrine-disrupting compounds may impact lipid metabolism. For example, a randomized controlled study in mice found that pubertal mice that were exposed to cypermeyhrin, 17 alpha-estradiol, and atrazine experienced a decrease in weight gain early in life [8]. Additionally, researchers found that early exposure to these compounds resulted in a significant decrease in the transcription of certain genes pertaining to T synthesis and cholesterol transport in the testes [8]. Another randomized controlled study conducted in mice suggested that exposure to pollutants, including parabens, upregulated several genes related to triglyceride deposition and adipose tissue triglyceride lipase [9]. In humans, levels of urinary concentrations of methylparabens, ethylparabens, and propylparabens were found to be significantly and inversely associated with obesity in adults and particularly females who were representative of the U.S. population [4].

To our knowledge, there are no studies associating urinary paraben levels with serum triglyceride concentrations in humans. Triglycerides are an ideal marker to measure urinary paraben levels because triglycerides are stored in adipocytes, where they can interact with parabens, and triglycerides are used in various aspects of lipid metabolism [6–9]. More importantly, this investigation is warranted because there seems to be a disconnect between the evidence generated from the studies with murine animal models versus the limited evidence generated from the few, small cross-sectional studies conducted in humans [1–4, 8, 9]. An important limitation of any study investigating parabens is that they have a relatively short half-life [1]. Additionally, the mechanism by which lipids interact with parabens is not fully understood [7]. Thus, the objective of this analysis was to evaluate the association between the concentrations of urinary parabens and triglycerides using data from NHANES.

## 2. Methods

### 2.1. Study Population

Study participants were enrolled in the National Health and Nutrition Examination Survey (NHANES). NHANES is a program that is affiliated with the Centers for Disease Control and Prevention and is tasked with assessing the health and nutritional status of Americans [12]. The primary objective of NHANES is to produce quality data that can be analyzed by health scientists to inform public health policy.

The study presented is a cross-sectional study that uses data collected by NHANES 2013-2014 [12]. It was a secondary analysis that examined biomarkers for their concentrations of urinary parabens and serum triglycerides. In order to be included in the analysis, participants needed to be at least 20 years old and have reported data on their triglyceride concentrations, each paraben concentrations, age, gender, race/ethnicity, and BMI. Under current regulations (§46.104), this study is considered exempt as determined by Florida International University's Institutional Review Board [13].

### 2.2. Assessment of the Exposure

The NHANES surveillance system measured urinary paraben concentrations in a third of the participants >20 years of age at a Mobile Examination Center [14]. Samples were frozen at −20°C, packaged, and then sent to the National Center for Environmental Health for testing [14]. The various parabens (methyl, ethyl, and propyl) were tested by using online solid phase extraction coupled to high-performance liquid chromatography-isotope dilution tandem mass spectrometry with peak focusing [15]. The samples underwent hydrolysis and deconjugation [15]. Then, they interacted with 0.1 M of formic acid to become acidified and their analytes were subjected to the above testing method [14, 15]. The acceptable calibration curve for urinary paraben concentrations had a coefficient correlation above 0.98 for the limit of detection [15]. The lower limit of detection was 1.0 *μ*g/L for methylparaben, 1.0 *μ*g/L for ethylparaben, and 0.1 *μ*g/L for propylparaben, and sample concentrations that fell below lower limit of detection were given an assigned value and divided by the square root of 2 [14]. Urinary paraben concentrations were reported in ng/mL in the NHANES analysis [14, 15]. To account for variations in urinary dilution, NHANES protocol requires urine measurements to be adjusted by urinary creatinine concentration [16]. In terms of accuracy, repeated analysis of synthetic urine spike with standard was calculated and labeled with isotopes, allowing for automatic recovery corrections to be conducted [15]. This study only analyzed the results of participants older than 20 years of age, as children may concentrate and metabolize parabens differently. Each paraben was considered as a continuous variable.

### 2.3. Assessment of the Outcome

The NHANES surveillance system collected samples from participants (>20 years old) who fasted for at least 8.5 hours, but not a full day, at a Mobile Examination Center, and the samples were then frozen at −30°C, packaged, and sent to the University of Minnesota for evaluation [17]. The method that was used to evaluate the triglyceride concentration is based upon the triglyceride undergoing hydrolysis to become a glycerol molecule [17]. The glycerol molecule then underwent oxidation to become hydrogen peroxide and dihydroxyacetone phosphate [18]. Finally, the hydrogen peroxide interacted with 4-aminophenazone and 4-chlorophenol via a peroxidase and created a red dyestuff that was proportional to the triglyceride concentration [17]. The triglyceride concentration was measured photometrically via Roche P and Roche Cobas 6000 chemistry analyzers [17, 18]. Triglyceride concentrations were reported in mg/dL and converted to mmol/L by multiplying by 0.01129 [17, 18]. In terms of quality control, two levels of control were run: one was from the sample pool and the other is an elevated abnormal commercial control sample [18]. From this subset of participants, only those older than 20 years of age and having paraben level results (*N* = 827) were included in the analyses. Serum triglyceride concentrations were analyzed as continuous variables.

### 2.4. Assessment of Confounders

The following variables were considered confounders in this secondary analysis: age, gender, race/ethnicity, and BMI [12, 19]. Age was assessed as continuous variable and only adults who are 20 years or older were included because triglyceride markers and possibly urinary paraben markers may differ in adults and children. Gender was categorized as males and females. Race/ethnicity was categorized as non-Hispanic Whites, non-Hispanic Blacks, non-Hispanic Asians, other Hispanics, Mexican Americans, and mixed race or others. Body mass index (BMI; kg/m^2^) was evaluated as continuous variables [12, 19].

### 2.5. Bias

Due to its low detection frequency (32%), butylparaben was eliminated from the study. The geometric mean of each paraben was calculated and compared to other NHANES publications. Adjusted and unadjusted results were generated to ensure quality control. Finally, NHANES has implemented quality control measures such as probability sampling designs to avoid sample bias [20]. Participant sample selections are representative of the United States population.

### 2.6. Statistical Analysis

Descriptive statistics (% for categorical variables and mean and standard deviation for continuous variables) were used to summarize participant's characteristics. Geometric mean, detection frequency %, and interquartile range were calculated to evaluate the distribution of the various parabens. Normality of the dependent variable was analyzed with the Shapiro–Wilk test, which showed that it was positively skewed; therefore, data were transformed using log10. Several models (adjusted and unadjusted) were constructed to analyze the association between each urinary paraben metabolite (methyl, ethyl, and propyl) and the triglyceride concentrations which used age, gender, race/ethnicity, and BMI, as predictors (in the adjusted models).

### 2.7. Simulated Models

1. Model 1: triglyceride concentrations and methylparaben (testing hypothesis I).
2. Model 2: predictors—age, gender and race/ethnicity, BMI, triglyceride concentrations, and methylparaben (testing hypothesis II).
3. Model 3: triglyceride concentrations and methylparaben (testing hypothesis III).
4. Model 4: predictors—age, gender and race/ethnicity, BMI, triglyceride concentrations, and ethylparaben (testing hypothesis IV).
5. Model 5: triglyceride concentrations and propylparaben (testing hypothesis V).
6. Model 6: predictors—age, gender and race/ethnicity, BMI, triglyceride concentrations, and propylparaben (testing hypothesis VI).

The models above determined the statistical significance of each cofounder and evaluated the possible effects each had on urinary paraben concentrations when predicting serum triglycerides. In this study, *P* values, corresponding 95% intervals (CI), and standardized coefficients are listed in Tables 1 and 2, which show the unadjusted and adjusted models, respectively. Significance testing used an *α*-level of 0.05, singled-tail tests. The goodness-of-fit was evaluated by adjusted *R*^2^. All analyses were done via SPSS version 23.

In terms of statistical rigor, linearity was examined via partial plot regressions. There was an independence of residuals, as evaluated by a Durbin–Watson statistic of 1.921 for methylparaben, 1.919 for ethylparaben, and 1.922 for propylparaben. The assumption for normality was assessed by a P-P plot, and homoscedasticity was reviewed by visual inspection of a plot of regression standardized predicted value versus regression standardized residuals [19]. The test for multicollinearity indicated a low level for methylparaben (VIF = 1.097), ethylparaben (VIF = 1.25), and propylparaben (VIF = 1.074); it was below 3 for all other variables. Cook's distance values were evaluated, and all were below 1 [21].

## 3. Results

A total of 827 participants were included in the analyses; their mean age was 49.7 years (20–80 years old); 52.4% were female and 47.6% were male (Table 3). Participants identified as 42.9% non-Hispanic Whites, 20.2% non-Hispanic Blacks, 13.1% non-Hispanic Asians, 20.8% Hispanics (12% Mexican American and 8.8% other Hispanics), and 3.0% mixed race or others. The average BMI was 28.9 (measured in kg/m^2^), which falls in the overweight category.

The geometric mean, detection frequency %, and interquartile range of methylparaben, ethylparaben, and propylparaben urinary concentrations were calculated from the single samples provided by the 827 participants (Table 4). The geometric mean was 57.1 ng/ml for methylparaben, 2.54 ng/ml for ethylparaben, and 6.54 ng/ml for propylparaben. Examining the previous reported data published by NHANES, methylparaben's geometric mean for those 20 years and above was reported to be between 60.3 and 63.0 in the years 2005–2010. For ethylparaben, the geometric mean for females and non-Hispanic Black in the years 2009-2010 was 4.63 and 2.62 (20 years and above was not reported), and propylparaben's geometric mean for those 20 years and above was reported to be between 7.58 and 8.67 in the years 2005–2010. The values reported are slightly lower than previous NHANES publications [22]. Methylparaben and propylparaben were detected at high frequency in urine samples, 99.3% and 98.8%, respectively; however, ethylparaben was only detected at a frequency of 50.1%. The limit of detection was 1.0 *μ*g/L for methylparaben, 1.0 *μ*g/L for ethylparaben, and 0.1 *μ*g/L for propylparaben.

The unadjusted result of each paraben tested and were found to be inversely associated with the experimental outcome variable, serum triglycerides concentrations; methylparaben (*β* =  −0.120, *P*=0.001), ethylparaben (*β* = −0.980, *P*=0.005), and propylparaben is (*β* = −0.104, *P*=0.003) (Table 1). The adjusted *R*^2^ for methylparaben was 0.013, ethylparaben was 0.008, and propylparaben was 0.010; *P* < 0.05 for all the models. Similar trends were reported when the model was adjusted for age, gender, BMI, and ethnicity and race.

In terms of the coefficients derived from the adjusted models (Table 2), methylparaben concentrations (*β* = −0.092, *P*=0.007), being female (*β* = −0.144, *P* < 0.001), non-Hispanic White (*β* = −0.135, *P*=0.011), and non-Hispanic Black (*β* = −0.253, *P* < 0.001) were associated with decreasing circulating triglycerides. Age (*β* = 0.099, *P*=0.003), being male (*β* = 0.144, *P* < 0.001), and BMI (*β* = 0.263, *P* < 0.001) were associated with increasing circulating triglycerides. The same significant trends were seen in the adjusted models for ethylparaben and propylparaben. The adjusted *R*^2^ for methylparaben was 0.125, ethylparaben was 0.122, and was propylparaben 0.123 (*P* < 0.05 for all the models). Taken together, these data suggest that each paraben has its own unique, albeit modest, influence on the various models.

## 4. Discussion

In these analyses, we examined the association between urinary paraben concentrations and serum triglyceride concentrations from NHANES dataset 2013-2014. We noted that there is a statistically significant inverse relationship between methylparaben, ethylparaben, and propylparaben and serum triglyceride concentrations. In addition, we observed that in relation to methylparaben, ethylparaben, and propylparaben, BMI, age, and being male were associated with increasing circulating triglycerides. Furthermore, being female, non-Hispanic White, and non-Hispanic Black were associated with decreasing circulating triglycerides in the presence of methylparaben, ethylparaben, and propylparaben. Finally, we noted the geometric mean of the study samples was comparable to previously reported data published by NHANES [22].

To our knowledge, no study has examined the association between urinary paraben concentrations and serum triglyceride concentrations. A cross-sectional study among adults representative of the U.S. population found that methylparaben, ethylparaben, and propylparaben were inversely associated with an increase in adiposity in adults [4]. Further, this study suggested that individuals who have larger deposits of adipose tissue may secrete less urinary paraben metabolites [4, 5, 23]. In terms of our study, this hypothesis would help explain why being female was associated with lower triglyceride concentrations when exposed to methylparaben, ethylparaben, or propylparaben and why those participants with a greater BMI did not have that association. It is also consistent with previous research that has found higher concentrations of various parabens in females [2, 3].

The key to understanding our results may lie in how each paraben is modulating various lipids. Flasiński et al. report that lipid modulating effects are associated with the length of the hydrocarbon portion of the ester molecule, suggesting that the more hydrophobic this section is, the larger an impact it has; butylparaben is the most hydrophobic of the four main parabens [7]. Their study in mammalian and bacterial lipid monolayers observes that butylparabens were disrupting and collapsing lipid monolayers at lower surface pressure [7]. Further, they suggest that methylparaben, ethylparaben, and propylparaben are interfacing with the different lipid monolayers, but to a lesser degree, with the ability to discriminate in their activity depending upon the phospholipid monolayer [7]. This is consistent with our results, as we found that methylparaben, ethylparaben, and propylparaben levels are associated with serum triglyceride levels. With the current evidence combined, we hypothesized that methylparaben, ethylparaben, and propylparaben may upregulate triglyceride metabolism in adipocytes if they interface at the proper receptor, orientation, and concentration. Although this would not explain all the discrepancies in the literature on parabens, it may explain some of it.

These analyses have some limitations and so the results should be interpreted accordingly. Our analyses relied on a single urine sample to predict chemical exposure in a compounds that has a relatively short half-life. Another issue is that this was a cross-sectional study, and therefore, this type of design does not allow us to infer causality. Finally, it is important to note that although the greatest association between methylparaben, ethylparaben, and propylparaben and lowering circulating triglycerides was seen among non-Hispanic Blacks, these data may be slightly skewed. People of African descent generally have lower circulating triglycerides, even though they may have other markers of lipid dysfunction [24]. Our study has several strengths worthy of note. To our knowledge, no study has examined the association between urinary paraben concentrations and serum triglyceride concentrations. Secondly, it used data generated from NHANES, which makes our results representative of the U.S. population. This study created working models that were able to analyze and control various covariates to determine statistical significance; however, these relationships need to be explored further in metabolic and mechanistic studies.

## 5. Conclusion

The geometric mean of the urinary concentration of methylparaben, ethylparaben, and propylparaben was lower, but in line with previous research. Further, the analyses of data from NHANES 2013-14 showed that methylparaben, ethylparaben, and propylparaben concentrations were significantly and inversely associated with triglyceride concentrations. These results suggest that the urinary excretion of methylparaben, ethylparaben, and propylparaben is associated with decreased circulating triglycerides. Further research is needed to confirm the mechanisms and health impact of this relationship.

## Figures and Tables

**Table 1:** Unadjusted results of each paraben with triglyceride concentrations (*N* = 827).

Model	*β*	*P*	95% CI of B
(1) Methylparaben	−0.120	0.001	0.000, 0.000
(2) Ethylparaben	−0.980	0.005	−0.001, 0.000
(3) Propylparaben	−0.104	0.003	0.000, 0.000

**Table 2:** Analysis of the coefficients of 2013-2014 NHANES participants (*N* = 827).

Model	*β*	*P*	95% CI of B
*(1) Methylparaben*	−0.092	0.007	0.000, 0.000
Age	0.099	0.003	0.001, 0.002
BMI	0.263	0.000	0.007, 0.012
Female	−0.144	0.000	−0.107, −0.040
Male	0.144	0.000	0.040, 0.107
Non-Hispanic White	−0.135	0.011	−0.123, −0.016
Non-Hispanic Black	−0.253	0.000	−0.221, −0.101

*(2) Ethylparaben*	−0.066	0.045	0.000, 0.000
Age	0.098	0.003	0.000, 0.002
BMI	0.256	0.000	0.007, 0.012
Female	−0.149	0.000	−0.109, −0.043
Male	0.149	0.000	0.043, 0.109
Non-Hispanic White	−0.132	0.013	−0.122, −0.014
Non-Hispanic Black	−0.266	0.000	−0.229, −0.110

*(3) Propylparaben*	−0.076	0.025	0.000, 0.000
Age	0.100	0.002	0.001, 0.002
BMI	0.264	0.000	0.007, 0.012
Female	−0.144	0.000	−0.107,−0.040
Male	0.144	0.000	0.040, 0.107
Non-Hispanic White	−0.128	0.015	−0.120,−0.013
Non-Hispanic Black	−0.256	0.000	−0.233,−0.103

**Table 3:** Demographic characteristics of the 2013-2014 NHANES sample participants (*N* = 827).

Variable	Mean ± SD or %
Age (years)	50.2 ± 17.4
BMI	28.9 ± 7.0
Gender	
Female	52.4%
Male	47.6%
Race/Ethnicity	
Non-Hispanic Whites	42.9%
Non-Hispanic Blacks	20.2%
Non-Hispanic Asians	13.1%
Mexican Americans	12.0%
Other Hispanic	8.8%
Mixed race or others	3.0%

**Table 4:** Distribution of methylparaben, ethylparaben, and propylparaben concentrations (ng/ml) among participants (*N* = 827).

Parabens (ng/ml)	Methyl	Ethyl	Propyl
(1) Geometric mean	57.1	2.54	6.54
(2) Detection frequency (%)	99.3	50.1	98.8
(3) Interquartile range	182.4	6.49	39.1
(i) 25%	17.5	0.71	1.10
(ii) 50%	55.4	1.00	6.30
(iii) 75%	199.9	7.20	40.2

*Note*. The limit of detection was 1.0 *μ*g/L for methylparaben, 1.0 *μ*g/L for ethylparaben, and 0.1 *μ*g/L for propylparaben.

## Data Availability

The NHANES dataset used to support the findings of this study have been deposited, but not made publicly available, in Mendeley Data, V1 repository https://doi.org/10.17632/f6d7pr8tf6.1. The following link will allow the readers to view and download the dataset: https://doi.org/10.17632/f6d7pr8tf6.1.
